# The Role of Whole Blood Transfusions in Civilian Trauma: A Review of Literature in Military and Civilian Trauma

**DOI:** 10.7759/cureus.24263

**Published:** 2022-04-18

**Authors:** Shane Kronstedt, Joon Lee, David Millner, Connor Mattivi, Halli LaFrankie, Lorenzo Paladino, Jeffrey Siegler

**Affiliations:** 1 Department of Medicine, Rutgers Robert Wood Johnson Medical School, New Brunswick, USA; 2 Department of Emergency Medicine, State University of New York Downstate Health Sciences University, Brooklyn, USA; 3 Department of Emergency Medicine, Washington University School of Medicine, St. Louis, USA

**Keywords:** surgery general, acute care surgery and trauma, emergency medicine resuscitation, fluid resuscitation, trauma resuscitation, hemorrhagic shock, trauma patients, massive blood transfusion, fresh whole blood, military trauma

## Abstract

Resuscitation techniques for the management of adult trauma patients have evolved over the 20th century. Whole blood transfusions were previously used as the standard of care, whereas blood component therapy is the current method employed across most trauma centers across the United States. Prior to the transition, no studies were conducted to show improved efficacy of hemostatic potential in trauma patients. Recent conflicts in Iraq and Afghanistan have challenged the dogma that whole blood transfusions are not the standard of care and have shown potential as the superior transfusion product for adult trauma patients. The purpose of this review is to provide a comprehensive review and elucidate if whole blood transfusions have a role in civilian trauma patients based upon recent military medical literature and civilian pilot studies using whole blood transfusions.

## Introduction and background

Historically, whole blood transfusions (WBTs) have been performed as a treatment for hemorrhagic shock since 1916. In World War II, whole blood transfusions were used successfully to treat hemorrhagic shock and were the preferred fluid of choice for resuscitation [1,2]. Improvements in blood storage and delivery, such as adding glycerol to red blood cells (RBCs) for freezing and delivery of blood products in plastic bags, were applied to battlefield medicine in the 1970s during the United States-Vietnam conflict and were among the numerous trauma medicine advancements from the period that became widely adopted in civilian medicine [3]. Due to the advancements in blood banking in the 1970s, blood has been broken down into components: red blood cells, platelets, and plasma for reasons including storage, shelf life, and the ability to give the separate components in specific diseases (e.g., administration of platelets in thrombocytopenia). However, no studies were conducted to show improved efficacy of hemostatic potential in adult trauma patients, civilian or military, prior to replacing WBT with blood component therapy [4].

Approximately 25,000 civilian deaths a year are attributed to preventable traumatic hemorrhagic shock in the prehospital phase of resuscitation in just the United States alone. Globally, this number climbs to as high as 1,000,000 in civilian settings. The availability of blood products in the prehospital resuscitation phase can improve morbidity and mortality, especially for patients with a transport time of over 30 minutes [5,6]. Shackelford et al. conducted a study on prehospital blood transfusions in military trauma patients in Afghanistan; a six-fold benefit in 24-hour mortality (p=0.001) and a three-fold benefit (Number Needed to Treat (NNT)<6) in 30-day mortality (p=0.005) were seen when red cells were received within 30 minutes of injury [7]. The most simple and effective blood product to use is group O whole blood; group O whole blood is particularly valuable globally in settings where optimal storage conditions and durations are more complex, making ABO-specific blood and titers more of a challenge [6].

While fluid resuscitation and hemorrhage control treatments have improved starkly, there remains room for improvement in the field. In an effort to improve the delivery of care to patients, there has been ongoing interest in the administration of blood components in different ratios to optimize morbidity and mortality outcomes [8]. As a result, there has also been a resurgence of interest in the use of WBT. Whole blood has the advantage of providing immediate access to life-saving red blood cells, plasma volume, and clotting factors; whole blood contains all of the preferred components of the recommended 1:1:1 ratio of trauma resuscitation that is traditionally used with blood component therapy (BCT). Additionally, the transfused RBCs have the potential to be younger than those provided by BCT under certain transfusion protocols [9]. The convenience and availability of point-of-care testing allow for a large donor pool. Sourcing from single donors per administration limits exposure to bloodborne diseases [10]. There is also the significant advantage of reducing the amount of citrate infused into the patient compared to equivalent blood components, as hypocalcemia in trauma patients is linked to an increase in mortality and has been shown to be low upon arrival in trauma patients [11,12]. Emerging literature in both the military and civilian sectors show that these hypothetical benefits have the potential to translate into measurable outcomes in the civilian sector. The purpose of this review is to highlight military data and civilian pilot studies to assess the recent measurable benefits of WBT and its application in civilian trauma patients.

## Review

Methods

A literature search was conducted to compare whole blood transfusions to component blood therapy in adult trauma patients to assess outcomes in 24-day, 30-day, and overall mortality. Using PubMed, Scopus, Cochrane Central, and ClinicalTrials.gov., articles were searched using search terms (Wounds and Injuries [Majr] OR Hemorrhage [Majr] OR Trauma [tiab] OR Injury [tiab] OR Injuries [tiab] OR Wound [tiab] OR Wounds [tiab]) AND ("Blood Transfusion"[Majr:NoExp] OR Whole blood transfusion [tiab]) AND ("Blood Transfusion"[Majr:NoExp] OR "Blood Component Transfusion"[Majr] OR Blood Component Transfusion [tiab]). Studies were selected based on the inclusion criteria: the population is adult trauma patients (aged above 18), only English language publications, randomized control trials, clinical trials, controlled clinical trials, retrospective studies, comparative studies, human study population, and study groups that used WBT. Studies were excluded if they were incomplete, clinical trial was ongoing, non-human study population, or report was unattainable.

**Figure 1:**
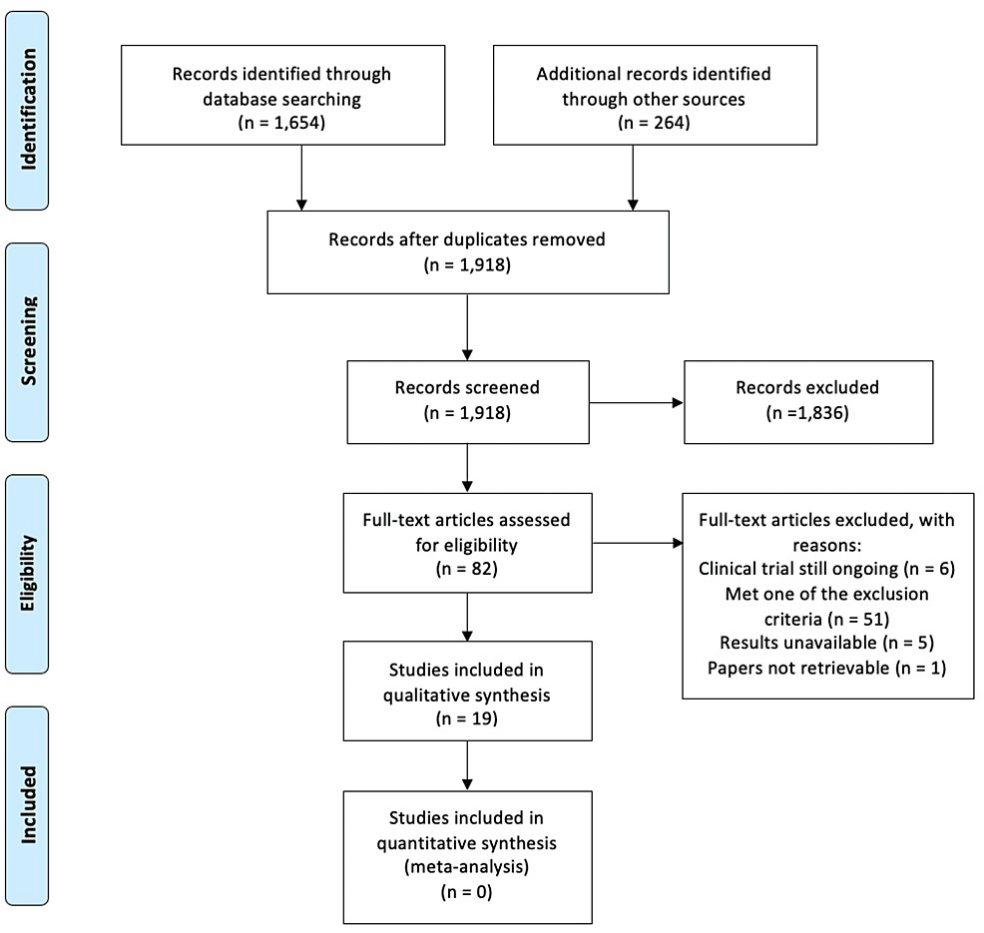
PRISMA flow diagram indicating study selection PRISMA: Preferred Reporting Items for Systematic Reviews and Meta-Analyses.

Initial articles were screened through title and abstract review for inclusion by author SK. Further screening and selection of articles for full-text review were completed by authors SK, HL, JL, DM, and JS independently. Authors SK, HL, JL, and DM conducted further qualitative data extraction. JS was available as an independent decision-maker if any disagreement occurred. Upon review, 1,918 articles were reviewed for inclusion (Figure 1). Ultimately, 19 articles were included for qualitative study in this paper per the inclusion and exclusion criteria outlined above. Since this paper was not intended to be a systematic review or meta-analysis, we did not assess for bias or level of evidence.

Results

Military Studies

The interest and adoption of WBT in the civilian setting were due to success during the United States military conflicts in Afghanistan and Iraq. A retrospective analysis by Nessen et al. (2009) of a split forward surgical team in military conflict in Afghanistan had a primary outcome following all-cause mortality of the teams and discussed secondary outcomes following transfusion practices and outcomes [13]. However, they documented no significant difference in WBT vs. other component therapies. However, this study is limited as comparing different transfusion therapies was not the primary outcome. Mortality in patients receiving a massive blood transfusion who received WBT was 19%, while other transfusion therapy, including BCT and factor VII transfusion, had a mortality rate of 26% [13]. In a later study, Nessen et al. (2013) conducted a retrospective cohort that examined fresh whole blood (FWB) use vs. BCT (packed red blood cells (pRBC) and fresh frozen plasma (FFP)) in combat casualties; they reported an odds ratio of 0.096 (95% CI=0.02-0.53) survival benefit in those who received RBCs, FFP, and WBT compared to those who received RBCs and FFP only [14]. Their findings showed no difference in mortality between non-group O recipients receiving group O whole blood compared to cross-matched WBT [14].

Spinella et al. (2009) reported a similar mortality benefit in their retrospective analysis [5]. The study contained a similar patient population of combat-injured US military soldiers and evaluated 24-hour and 30-day mortality of those who received RBCs and WBT compared to RBCs and FFP only. At 24 hours, the WBT cohort had a survival of 96% (n=100) compared to 88% (n=254) in the BCT cohort (p=0.018). At the 30-day comparison, the WBT had a 95% survival compared to the BCT cohort of 82% (p=0.002). Injury Severity Scores (ISSs) were comparable between the cohorts [5].

In a retrospective cohort released by Perkins et al. (2011), they reported that in combat trauma patients receiving resuscitation with apheresis platelets without any FFP, RBCs (n=284), or WBT (n=85), there was no mortality benefit at 24 hours (84% vs. 81%, p=0.52, respectively) or 30 days (60% vs. 57% respectively, p=0.72) [15]. While the authors state that the use of WBT in the routine management of civilian trauma requires additional research, they nonetheless conclude that FWB could serve as an adequate source of platelets in conditions where apheresis platelet products are scarce [15]. Gurney and colleagues (2022) used the Joint Trauma System Role 2 Database in retrospective cohort analysis to determine how WBT vs. BCT affected six-hour mortality in US military casualties in Afghanistan from 2008 to 2014 [16]. Their study reported two cohorts, warm fresh whole blood (n=221) and non-warm fresh whole blood (n=884). It was stratified based on different variables such as injury type, tourniquet use, prehospital transfusion, and transfusion rate. They reported a statistically significant reduction in six-hour mortality among the WBT group vs. BCT (OR=0.27, CI=0.13-0.58), with an even further reduction in mortality when adjusting for the aforementioned variables (OR=0.15, p=0.24) [16].

In a retrospective study of battle injuries presented to three US Marine Corps surgical facilities in Afghanistan from January 2010 to July 2012, Auten et al. described comparisons in a BCT cohort (n=35) vs. BCT augmented with WBT (n=26) [17]. They noted that the cohort augmented with WBT had a higher average ISS (30.2 for BCT, 25.3 for BCT augmented with WBT, p=0.12). However, they had a small sample size, in which each cohort had two deaths and showed no statistical difference in 24-hour or 30-day mortality (OR=0.81; 95% CI=0.08-8.842) [17].

In a retrospective study that reviewed trauma patients admitted to the Combat Support Hospital in Ibn Sina Hospital in Baghdad, Iraq, from January 2004 to December 2006, Chan et al. (2012) reviewed the incidence of acute lung injury (ALI) in patients who received transfusions with or without fresh whole blood [18]. Among 591 patients, they found that patients who had developed ALI (11.2% of the total population) were more likely to have received WBT (34% in WBT vs. 20% in non-WBT, p=0.1). However, they note confounding variables such as increased injury and crystalloid use in patients receiving whole blood. Their total mortality outcomes were 41.5% in whole blood groups vs. 45.6% in non-whole blood groups (p=0.466) [18].

Keneally and colleagues (2015) conducted a retrospective analysis using the Joint Theater Trauma Registry, analyzing patients at US military facilities in Afghanistan and Iraq presenting for thoracic trauma from 2002 to 2012 [19]. They compared patients transfused with component therapy augmented with whole blood (n=281) vs. BCT only (n=3656). While initial results showed an increase in mortality in patients receiving WBT (21.3% vs. 12.8%, p≤0.001), once the results were adjusted for confounding variables, they found that there was no statistically significant increase in mortality (OR=1.247, 95% CI=0.760-2.048, p=0.382) [19].

In a review of fresh whole blood transfusions in military practice, Kauvar et al. (2006) analyzed transfusion practices in Operation Iraqi Freedom between March and December 2003 [20]. They report that a total of 2,349 blood products were transfused to 281 patients; they note that there was no statistical difference (p=0.44) in mortality between the WBT group (n=36, mortality=19%) and the BCT group (n=230, mortality=12%). Although no statistical differences in mortality were found, they note the logistical difficulties in using BCT in combat operations and conclude that WBT is a safe and effective method in saving the lives of patients who might otherwise have died [20].

Civilian Studies

In a civilian pilot study by Cotton and colleagues (2013), they created a randomized controlled study design that included 107 patients randomized to receive WBT or BCT [21]. This resulted in 55 WBT patients and 52 BCT patients per intent-to-treat analysis, and there was no difference in 24-hour and 30-day mortality (p=0.83, p=0.26, respectively); 39 WBT and 42 BCT per-protocol analyses showed no significant difference in 24-hour and 30-day mortality between the two groups (p=0.58, p=0.16, respectively). Of note, there was a disparate amount of traumatic brain injury (TBI) seen in patients enrolled in the WBT cohort despite randomization; TBIs accounted for most of the causes of death in the WBT group, and exsanguination was the leading cause of death in the BCT cohort. In a sensitivity analysis of the WBT cohort, they noted that WBTs significantly reduced the amount of total products received; there was a strong association with decreased platelet transfusions for those who received a WBT (p=0.09) [21].

Jones and Frazier (2014) conducted a secondary data analysis of the National Trauma Data Bank [22]. After controlling for variables such as age, gender, and emergency medical service (EMS) transfer time, they concluded that those who were transfused with BCT (n=1,662) had increased odds of mortality (OR=3.2; 95% CI=1.314-7.618; p=0.010) when compared to the WBT group (n=83), despite having an identical ISS. The authors note that the significantly larger sample size in the BCT group vs. the WBT group underlines how the use of BCT has become ubiquitous in typical transfusion practices, but the WBT group demonstrates promise as a possible superior alternative to BCT in reducing mortality [22].

Additional studies have discussed the use of Low Titer type O negative WBT (LTOWB) in US-based trauma centers with a primary outcome of hemolysis studies and secondary outcomes of survival and blood product usage. Williams et al. (2020) used a retrospective cohort design where WBT (n=198) was compared to BCT (n=152) [23]. After controlling for age, injury severity, blood pressure, and pH, the use of WBT was found in a multivariate analysis to be an independent predictor for increased 30-day survival (OR=2.19; 95% CI=1.010-4.767; p=0.047) and 53% reduction in post-emergency department transfusion of blood products (OR=0.47; 95% CI=0.23-0.94; p=0.033). The primary outcome of hemolysis showed no significant difference through 48 hours based on numerous variables analyzed [23].

In a prospective observational study by Shea and colleagues (2020), they measured the primary outcome of 24-hour mortality in trauma patients aged 18 and older with massive transfusion protocol (MTP) activations [24]. By comparing the data between the BCT (n=42) and the LTOWB (n=44) groups, the authors indicated that WBT use is independently associated with both improved survival odds in both 24-hour and 28-day survival (p<0.001). Specifically for 24-hour mortality, the authors noted an improved odd of survival by 23% in the WBT group (OR=0.81; 95% CI=0.69-0.96; p=0.017), whereas, for 28-day mortality, they noted a statistically significant improved survivability in the WBT group (HR=0.30; 95% CI=0.14-0.65; p=0.002) [24].

Seheult et al. (2018) performed a retrospective review of trauma patients who received LTOWB (n=135) compared to matched traditional BCT (n=135) and showed no significant difference in the primary outcome of in-hospital mortality (p=0.24), 24-hour mortality (p=0.33), and ICU length of stay (p=0.16) [25]. Secondary outcomes of the number of units transfused were not statistically significant. One possible explanation as to why no difference was seen in the recipients who received LTOWB was that they received a relatively small number of transfusions compared to their conventional component counterparts; the median amount of blood received by the LTOWB group was two units; this is approximately equal to only one half of the typical adult dose of plasma or platelets. This quantity is unlikely to show a significant hemostatic effect. Normalization of lactate levels in the WBT group was shorter than standard BCT therapy [25].

Rahbar and colleagues (2015) conducted a randomized controlled trial that further analyzed the laboratory findings of WBT vs. BCT [26]. The trial demonstrated similar results that may further indicate improved physiologic function with WBT compared to BCT. The pilot study showed that WBT and BCT declined in initial thromboelastographic parameters at three and six hours. However, patients receiving WBT exhibited improved thrombin potential and platelet aggregation to ristocetin. No data were reported on improved morbidity or mortality associated with these laboratory findings [26].

In a retrospective review of a Level 1 Civilian Trauma Center Registry, Kemp Bohan et al. (2021) reported no significant difference in 24-hour mortality when comparing WBT to BCT [27]. Two hundred sixteen patients admitted with hemorrhagic shock receiving blood products prehospital or within 24 hours were grouped into three cohorts receiving either WBT (n=34), BCT (n=95), or WBT plus BCT (n=87). There were no significant differences across the three study groups regarding mortality (p=0.45); however, at 30 days and during the hospital stay, mortality was higher in the WBT+BCT cohort vs. the WBT only and BCT groups; however, this was not shown to be statistically significant (p=0.05 and p=0.06, respectively) [27].

A single-center, prospective cohort pilot study by Siletz and colleagues (2021) compared secondary outcomes of mortality, morbidity, intensive care unit stay duration, and hospital-free days between trauma patients resuscitated with WBT+BCT (n=38) vs. BCT only (n=32) [28]. The study showed no differences in morbidity outcomes, and the differences in mortality (4.4% for WBT plus BCT vs. 11.7% for BCT only) were not significant (p=0.19) [28].

A single-center, retrospective study by Yazer and colleagues (2021) analyzed trauma patients receiving at least three units of WBT (n=155) vs. BCT (n=165) within the first 24 hours of admission and compared clinical outcomes [29]. The authors ultimately concluded that there were no significant differences in six-hour (p=1.000), 24-hour (p=0.734), and 30-day mortality (p=0.582); there was no difference in the frequency of other clinical outcomes between the cohorts, except for a lower delta multiorgan dysfunction score in the WBT group (p=0.039) [29].

Braverman et al. (2021) investigated a single institutional trauma registry for outcomes of patients based on prehospital transfusion (PHT) of LTOWB (n=107) vs. no transfusion (NT) (n=431) [30]. Among many outcomes, they found no statistically significant difference in 24-hour death (p=0.6) or all mortality (p=0.25). However, they note that patients receiving PHT had a more significant reversal of shock upon arrival (-0.28 PHT vs. -0.002 NT, p<0.001), suggesting a role for WBT in the prehospital setting [30].

Mortality and Outcomes

Of the 19 articles, 15 were retrospective cohort studies, two were prospective cohort studies, and two were randomized control trials. One of the retrospective studies sampled data from the National Trauma Data Bank, and the other articles were all collected from single-center Level 1 Trauma Civilian and Military Centers’ registries. Overall, improved 24-hour survival and 30-day survival were seen with clinical significance and statistical significance in multiple studies for patients receiving WBTs, showing potential as a superior transfusion product (Tables 1, 2). The benefit seen for all mortality (Table 3) showed a similar theme. Some studies showed a statistically significant difference, and others showed a mortality benefit, but the findings were not considered statistically significant.

**Table 1:** Comparison of 24-Hour Mortality Outcomes in WBT vs. BCT WBT: whole blood transfusion; BCT: blood component therapy.

Author	Experiment vs. Control	24-Hour Mortality
Spinella et al. (2009) [5]	WBT vs. BCT	Lower mortality in the WBT group (p=0.018)
Perkins et al. (2011) [15]	WBT vs. BCT	No significant difference (p=0.52)
Auten et al. (2015) [17]	WBT vs. BCT augmented with WBT	No significant difference (OR=0.81; 95% CI=0.08-8.842)
Cotton et al. (2013) [21]	WBT vs. BCT	No significant difference
Shea et al. (2020) [24]	WBT vs. BCT	Improved survival in the WBT group by 23% (HR=0.15; p=0.017)
Seheult et al. (2018) [25]	WBT vs. BCT	No significant difference (p=0.33)
Kemp Bohan et al. (2021) [27]	Arm 1: WBT vs. WBT+BCT	No significant difference between all three cohorts (p=0.45)
Kemp Bohan et al. (2021) [27]	Arm 2: BCT vs. WBT+BCT	No significant difference between all three cohorts (p=0.45)
Yazer et al. (2021) [29]	WBT vs. BCT	No significant difference
Braverman et al. (2021) [30]	WBT vs. no transfusion	No significant difference (p=0.6)

**Table 2:** Comparison of 30-Day Mortality Outcomes in WBT vs. BCT WBT: whole blood transfusion; BCT: blood component therapy.

Author	Experiment vs. Control	30-Day Mortality
Spinella et al. (2009) [5]	WBT vs. BCT	Lower mortality in the WBT group (p=0.002)
Perkins et al. (2011) [15]	WBT vs. BCT	No significant difference (p=0.72)
Auten et al. (2015) [17]	WBT vs. BCT augmented with WBT	No significant difference (OR=0.81; 95% CI=0.08-8.842)
Cotton et al. (2013) [21]	WBT vs. BCT	No significant difference
Shea et al. (2020) [24]	WBT vs. BCT	Improved survival in the WBT group (p<0.001)
Yazer et al. (2021) [29]	WBT vs. BCT	No significant difference

**Table 3:** Comparison of All Mortality Outcomes in WBT vs. BCT WBT: whole blood transfusion; BCT: blood component therapy; FWB: fresh whole blood; PHT: prehospital transfusion; CBT: component blood therapy; N/A: not applicable.

Author	Experiment vs. Control	All Mortality
Nessen et al. (2009) [13]	WBT vs. other transfusion therapy in military conflict	All mortality: WBT=19% vs. CBT=26% (p=0.87)
Nessen et al. (2013) [14]	WBT vs. BCT	Improved survival with patients who received FWB vs. BCT (95% CI=0.02-0.53)
Gurney et al. (2022) [16]	WBT vs. BCT	Statistically significant reduction in six-hour mortality among the WBT group vs. BCT (OR=0.27; 95% CI=0.13-0.58) with an even further reduction in mortality when adjusting for injury type, tourniquet use, prehospital transfusion, and transfusion rate (OR=0.15; p=0.24)
Chan et al. (2012) [18]	WBT vs. non-WBT transfusions	No significant difference (p=0.466)
Keneally et al. (2015) [19]	WBT vs. BCT	Initial results showed an increase in mortality in patients receiving WBT (21.3% vs. 12.8%, p≤0.001); however, once the results were adjusted for confounding variables, there was no statistically significant increase in mortality (OR=1.247, 95% CI=0.760-2.048, p=0.382)
Kauvar et al. (2006) [20]	WBT vs. BCT	No significant difference (p=0.44)
Jones and Frazier (2014) [22]	WBT vs. BCT	BCT with mortality OR=3.2 (p=0.010, 95% CI=1.314-7.618)
Williams et al. (2020) [23]	WBT vs. BCT	WBT with a two-fold increase in the likelihood of survival (95% CI=1.01-4.76; p=0.047)
Seheult et al. (2018) [25]	WBT vs. BCT	No significant difference in in-hospital mortality (p=0.24)
Rahbar et al. (2015) [26]	WBT vs. BCT	N/A; WBT patients exhibited improved thrombin and platelet aggregation to ristocetin. No data reported on improved morbidity or mortality associated with the findings
Kemp Bohan et al. (2021) [27]	Arm 1: WBT vs. WBT+BCT	No significant difference between WBT only vs. WBT+BCT (p=0.05)
Kemp Bohan et al. (2021) [27]	Arm 2: BCT vs. WBT+BCT	No significant difference between BCT only vs. WBT+BCT (p=0.06)
Siletz et al. (2021) [28]	WBT+BCT vs. BCT only	No significant difference (p=0.19)
Braverman et al. (2021) [30]	WBT vs. no transfusion	No significant difference (p=0.25), albeit patients receiving PHT had a greater reversal of shock upon arrival (p<0.0001)

Discussion

Current literature suggests that WBT shows promise as a treatment strategy for adult trauma patients in prehospital and hospital-based resuscitation due to traumatic hemorrhage. Pilot programs at civilian trauma centers are already underway across the United States to further research and validate this treatment strategy and its potential benefits. For example, a regional trauma network in Southwest Texas uses whole blood transfusions in their ground- and air-based EMS units. They report success in deploying their new program, especially in rural areas where access to a trauma center exceeds 60 minutes by ground. Their published data set is currently 25 patients. The results look promising, as they show a marked decrease in mortality (36% adults and 20% pediatrics vs. 60% overall mortality) compared to their historic rate using component therapy or crystalloid solutions. Additionally, they had zero transfusion reactions or seroconversions. Based on their initial success, they have begun implementing a more comprehensive system approach [31].

Before WBT is implemented on a large scale for the routine treatment of hemorrhagic resuscitation, logistical considerations should include changes to current blood product storage used at civilian hospitals that are designed for BCT. A recent investigation into whole blood storage shows that shelf life may be more limited than previously thought. Platelet count and function in stored whole blood decreased after seven days and 14 days [32]. Compared to components, whole blood therapy's shelf life can be extended depending on how it is prepared, and the whole blood units can be recycled into pRBCs in some areas.

As interest in WBT continues to expand into the civilian medical world, the potential uses for whole blood are also growing. For example, a recent pilot program treating obstetric hemorrhage secondary to placental accreta showed similar potential benefits in traumatic losses, including decreased transfusion volume needed and decreased post-transfusion complications [33]. Expanding research funding and whole blood access can continue to increase the use and expand the role of whole blood into different disciplines.

Limitations

Among the limitations of this study was the quality and power of the evidence. Of the 19 articles reviewed, 15 were retrospective cohort studies with two prospective cohort studies and two randomized control trials. Many studies referenced have low sample sizes, and the populations studied were primarily military casualties, which create limitations in the generalizability to typical civilian hospital systems and their populations. As noted in Kemp Bohan et al. (2021), another consideration is potential differences in the survivability of the wounds between the WBT and BCT therapies [27]. Differences in injury severity could have impacted the observed mortality comparisons between the various cohorts. Additionally, how injury severity may have played a role in how trauma providers determined which treatment protocols the patients received may demonstrate further confounding variability in the results [24]. In contrast, some wounds were not survivable and may or may not have reflected a failure in the treatment administered. Because we did not intend to perform a systematic review and meta-analysis, we did not assess for bias, level of evidence, or perform a pooled analysis.

## Conclusions

WBTs have an evolving body of evidence to support their use in traumatic hemorrhagic shock compared to BCT, albeit the current standard of care favors the use of BCT presently. However, this was done without sufficient evidence demonstrating that BCT was superior to WBT. Therefore, recommendations should be updated to reflect more recent studies comparing mortality outcomes of the two therapies in civilian trauma.

This review shows that studies using WBTs demonstrated non-inferiority or superiority, with variable statistical significance. The use of whole blood as a transfusion product has been shown to be both safe and effective and reduces the incidence of transfusion-induced hypocalcemia compared to citrate-containing BCT. Additionally, the availability of whole blood products shows reduced mortality in prehospital trauma, particularly for those with transport time greater than 30 minutes. This is especially pertinent considering the rates and sheer numbers of mortality attributed to preventable traumatic hemorrhagic shock in the prehospital phase of resuscitation in the United States and globally. The authors conclude that whole blood may be the superior product for adult trauma patients compared to component transfusions, with potential in the civilian space.

Most of the studies conducted were cohort studies that had limitations in their ability to control for confounding variables, such as the degree of injury and how that affected the patients' transfusion protocol. These confounders have the potential to have a profound impact on apparent differences in outcomes. Due to the difficulties in conducting randomized control trials in transfusion protocols for trauma, clinical data are limited and more studies are needed to better demonstrate more concrete associations on how these transfusion protocols will affect clinical outcomes.
